# Exploring Lacrimal Gland Tear Production in Sheep under General Anesthesia: Examining the Potential Impact of Utilizing 1% Hyaluronic Acid Ophthalmic Gel

**DOI:** 10.3390/life14081038

**Published:** 2024-08-20

**Authors:** Ruxandra Pavel, Ioana Ene, Ruxandra Costea

**Affiliations:** Faculty of Veterinary Medicine, University of Agronomic Sciences and Veterinary Medicine of Bucharest, 011464 Bucharest, Romania; ioana.ene@fmvb.usamv.ro (I.E.);

**Keywords:** sheep, anesthesia, lacrimal gland, tear production, ophthalmic gel

## Abstract

The aim of the study is to assess the lacrimal gland tear production, using the Schirmer Tear Test (STT), in healthy sheep under general anesthesia and to explore the effects of applying 1% hyaluronic acid ophthalmic gel during general anesthesia. While STT values during anesthesia have been well documented in small animals such as cats and dogs, there seems to be a lack of information available for ruminants like sheep. This gap in the literature highlights the need for further research and exploration into tear production in sheep under anesthesia. The experimental research conducted on twelve adults female sheep provided valuable insights into tear production under anesthesia. By assessing tear production at various key time points the study was able to capture the changes in tear production throughout anesthesia and revealed a significant decrease in Schirmer Tear Test values in all sheep, following general anesthesia. The results showed that closing and taping the eye yielded determined better outcomes compared to administering a 1% lubricant ophthalmic gel. This finding suggests that eye care during anesthesia can impact tear production in sheep.

## 1. Introduction

Tears play a vital role in preserving eye health by eliminating foreign particles, supplying crucial nutrients to the avascular cornea, and containing immunoglobulins, lysozymes, and other essential proteins that help defend the eye [1].

Histologically, the lacrimal glands consist of acini, which comprise layers of myoepithelial cells with lightly stained secretory granules, along with prominent serous cells. These structures are encased in a rare and vascular stroma [2].

When discussing eye examinations, it is crucial to follow specific steps to ensure there is no compromise and no further complications arise [3]. The Schirmer Tear Test (STT) is a crucial examination in ophthalmology for assessing tear production and diagnosing various eye pathologies like keratitis or keratoconjunctivitis sicca [4]. It plays a significant role in determining the quality and quantity of tears, providing valuable insights into the overall ocular health of an individual.

Insufficient tear production leads to keratoconjunctivitis sicca (KCS), commonly referred to as “dry eye” or xerophthalmia, causing gradual inflammation of the cornea and conjunctiva [1,5,6]. Ocular discharge, because of this inflammation, is a common symptom observed in individuals with KCS, although the exact extent of its increase may vary and remain uncertain. Hence, understanding the baseline tear production in healthy sheep is crucial, and this can be performed by the STT, currently considered the most accurate method to measure tear production in animals [7].

Tear film is composed of three layers which are produced by the meibomian glands and the glands of Zeis that form the outer thin and fatty layers that prevent the underlying aqueous layer from evaporating or overflowing [8]. The middle aqueous layer, which constitutes over 60% of the entire tear film, is primarily responsible for its function and is the thickest among the three layers. The lacrimal gland, accessory lacrimal glands, and the nictitans gland are the ones that produce the aqueous layer [9]. The main function of the aqueous layer of the tear film is represented by preserving nutrition and oxygen supply to the cornea along with the protection of its surface from foreign bodies, epithelial waste, or other toxic substances [10]. Conjunctival goblet cells produce the inner mucous layer that has its main characteristic of transforming the hydrophobic epithelial surface into a hydrophilic one [11].

Corneal abrasions are one of the most common ophthalmological complications related to general anesthesia during surgeries that do not involve the eye. It is important to be aware of this risk and take necessary precautions to prevent it [12]. Other complications may include lagophthalmos, resulting in exposure keratopathy, diminished eyelid reflex, lowered basal tear production, and compromised stability of the corneal tear film [13].

Anesthesia is frequently performed in sheep and pigs for experimental research, employing a diverse approach to ensure effective sedation [14,15].

The effect of anesthetics on tear production is evaluated in different species, but in sheep, the current veterinary literature has few studies regarding normal values of tear production and how general anesthesia affects the STT measurements [16].

Reduced tear production may result from the interaction of general anesthetics with the parasympathetic nervous system. Alternatively, it could be due to diminished blood flow in the lacrimal gland or alterations in the tear-producing cells [17].

In our study, we aimed to compare tear production in sheep during anesthesia using two different protection methods: taping the eye vs. applying a 1% hyaluronic acid ocular gel. While we did not come across any specific research on eyelid closure in sheep during anesthesia, there are several studies in human medicine that delve into the causes of corneal injuries linked to anesthesia and assess the advantages and disadvantages of various corneal protection methods [18,19,20,21,22,23].

The primary hypothesis posits that general anesthesia leads to a significant decrease in tear secretion. The secondary hypothesis suggests that closing the eyelid during anesthesia will help prevent rapid evaporation of the tear film and facilitate a quicker return to normal values of STT compared to situations where 1% lubricant ophthalmic gel was used without other protection.

## 2. Materials and Methods

This study was in accordance with the Ethics Committee of the Faculty of Veterinary Medicine Bucharest and the methodology used adhered to the guidelines outlined in the Public Health Service Policy on the Humane Care and Use of Laboratory Animals (2015). The study was conducted on 12 Tsurcana female sheep (24 eyes) during the spring season, with a mean age and weight of 6.33 years old and 48.25 kg, respectively. All animals were housed under the same environmental, nutritional, and welfare conditions. A thorough and calm preanesthetic physical examination was performed, evaluating cardiovascular and respiratory functions, temperature, and hydration status. The color of the mucous membranes was assessed, and additionally blood samples were obtained to assess health status of the sheep, including a complete ophthalmologically exam. All sheep included in this study were clinically healthy.

Patient preparation included a 12 h fasting and a 6 h water restriction. Regurgitation is more likely if this preoperative fast period is not followed. Fasting before anesthesia helps minimize gas production from fermentation and prevents rumen bloating. This, in turn, reduces intraabdominal pressure on the diaphragm and major blood vessels, thereby preventing compromised cardiovascular function [14].

**Figure 1:**
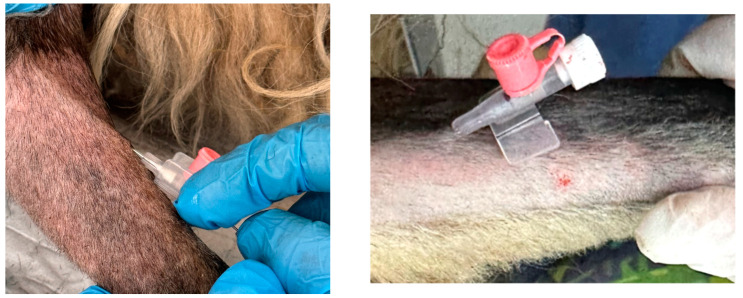
Sheep with a 20 G catheter in the cephalic vein.

All sheep were premedicated with 0.2 mg/kg of midazolam (Midazolam SUN 1 mg/mL, Sun Pharmaceutical Industries Europe B.V., Cluj-Napoca, Romania), 5 mg/kg of ketamine (Ketamidor^®^ 100 mg/mL, VetViva Richter GmbH, Wels, Austria), and 0.1 mg/kg of butorphanol (Butomidor^®^ 10 mg/mL, VetViva Richter GmbH, Wels, Austria) intramuscularly (IM). After premedication, a 20 G catheter was placed in the cephalic vein (Figure 1). The induction was performed with 3–6 mg/kg of propofol (Fresofol^®^ 1% MCT/LCT, Fresenius Kabi Australia, Mount Kuring-gai, Australia) intravenously (IV).

**Figure 2:**
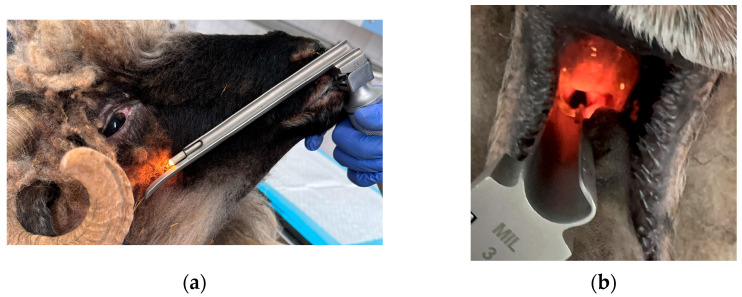
(**a**) Intubation using a laryngoscope with a Miller blade; (**b**) visualization of the larynx.

Sheep were intubated and maintenance of anesthesia was performed with isoflurane (Isothesia, 1000 mg/g, Piramal Critical Care B.V., Voorschoten, The Netherlands) and oxygen 100% (Figure 2a,b).

**Figure 3:**
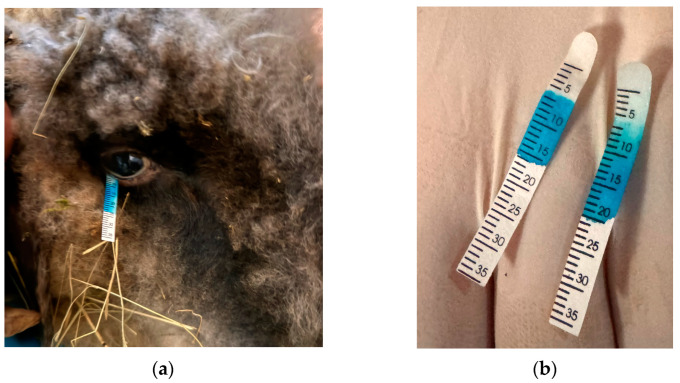
(**a**) Placing the Schirmer Tear Test; (**b**) values of the Schirmer Tear Test.

Schirmer Tear Test (Schirmer-Tränentest, Vet Eickemeyer^®^, Tuttlingen, Germany) was performed for both eyes, with baseline values being recorded and expressed in millimeters per minute. During STT, the eyelids were gently closed, and the test strip was placed into the temporal third of the lower eyelid for a duration of 1 min (Figure 3a). Following the removal of the test strip, the length of the moistened region was promptly measured in millimeters (Figure 3b).

Tear production was measured at baseline T_0_ (15 min before premedication). After 15 min of premedication, we recorded a second value of the STT (T_1_).

For the third value of the STT, immediately after intubation of the sheep, we chose to tap the eyelids of the right eye (OD) with adhesive band, and for the left eye (OS), we instilated 2 drops of a lubricant ophthalmic gel with 1% hyaluronic acid and waited 15 min before measuring the tear secretion (T_2_).

At the end of the surgery, we waited 15 min after extubation and we recorded a fourth STT value (T_3_). The last measurement was performed for all sheep 24 h after the end of the surgery (T_4_).

**Figure 4:**
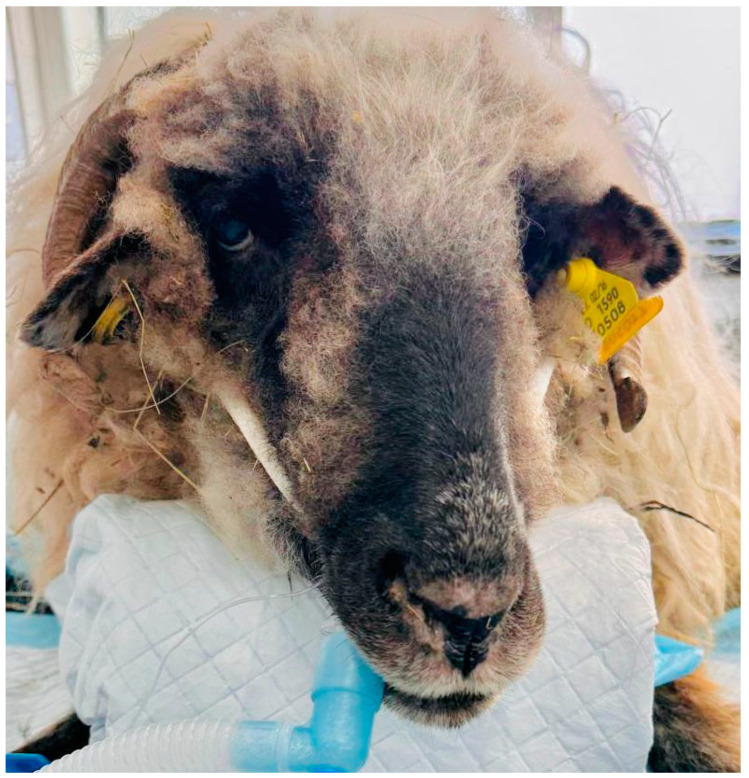
Head position during surgery, immediately after intubation.

The surgery of the sheep was represented by removing an electrode and electronic modules implanted subcutaneously in the right posterior leg, in the croup region, in a previous surgery. For this study, all sheep were positioned in sternal recumbency with the head straight (Figure 4).

All sheep had the same period of being under anesthesia, respectively, 1 h.

A statistical analysis of the data was performed using the software program DATAtab (DATAtab Team 2024. DATAtab: Online Statistics Calculator. DATAtab e.U. Graz, Austria. URL https://datatab.net, accessed on 12 July 2024) and Microsoft Excel (Version 16.82, 2024).

Mean values, along with the standard deviation (SD), were calculated for the STT results. All measurements were conducted around the same time of the day, between 9 am and 3 pm, to account for potential diurnal variations in tear production [24,25]. Additionally, the measurements were performed by the same individual.

A paired sample *t*-test was used to compare the STT values obtained before and after premedication, after taping the right eye (OD) and applying 2 drops of the 1% hyaluronic acid lubricant ophthalmic gel in the left eye (OS), after extubation, and after 24 h. We analyzed whether taping the eye without applying the ocular lubricants can influence the evaporation of the tear film and the return of the normal values faster. Statistical significance was defined as a *p*-value < 0.05.

## 3. Results

All measurements were completed successfully. It is known that during summer and spring, sheep tend to have higher STT values compared to winter, as described by Dedousi [26]. Previous research in horses and ponies has shown a tendency for increased tear scores in the STT during winter compared to those observed in summer, although these differences did not reach statistical significance [27]. Moreover, a previous study carried out on horses stated that the amount of tear secretion can vary according to the seasons [27]. All data were obtained in the spring season, within the same period of the day, from 9 am to 3 pm; therefore, changes in the daily cycle and season, which might influence the test results, most likely did not affect our STT results since the differences between STT values were observed in different seasons readings, early in the morning or late evening [4,24,28,29].

**Figure 5:**
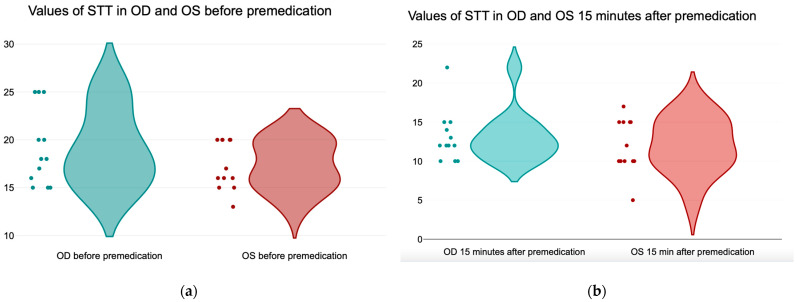
(**a**) Values of the Schirmer Tear Test in the right (OD) and left eye (OS) before premedication; (**b**) values of the Schirmer Tear Test in the right and left eye 15 min after premedication.

The mean values ± SD of basal tear production, expressed in millimeters/min, measured before premedication (T_0_) on right (OD) and left eye (OS), were 19.08 ± 3.96 mm/min and 17.33 ± 2.53 mm/min, respectively (Figure 5a). The mean values ± SD in both eyes after 15 min of premedication (T_1_) were 13.08 ± 3.31 mm/min for the right eye (OD) and 12 ± 3.43 mm/min for the left eye (OS) (Figure 5b).

**Figure 6:**
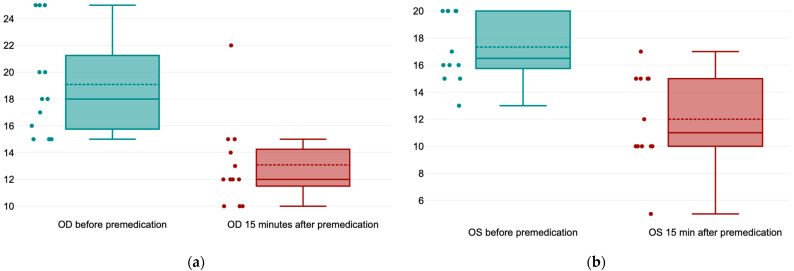
(**a**) Values of the Schirmer Tear Test in the right eye (OD) before premedication and 15 min after premedication; (**b**) values of the Schirmer Tear Test in the left eye (OS) before premedication and 15 min after premedication.

The results of the *paired-t* test indicated that there is a significant large difference between the right eye (OD) before premedication (T_0_) (*M* = 19.08, *SD* = 3.96) and after 15 min of premedication (T_1_) (*M* = 13.03, *SD* = 3.31), *t*(11) = 8.8, *p* < 0.001 (Figure 6a). Regarding the left eye (OS), the results of the paired-*t* test indicates that there is a significant large difference between the STT values before premedication (T_0_) (*M* = 17.33, *SD* = 2.53) and 15 min after premedication (T_1_) (*M* = 12, *SD* = 3.43), *t*(11) = 7.7, *p <* 0.001 (Figure 6b).

The mean values ± SD 15 min after intubation and taping the right eye (OD) and after instilating two drops of ocular gel with 1% hyaluronic acid in the left eye (OS) (T_2_) were 8.75 ± 2.70 and 3.58 ± 2.71 mm/min, respectively. The *t*-value is 4.67584, and the *p*-value is 0.000058. The result is *significant* at *p* < 0.05.

**Figure 7:**
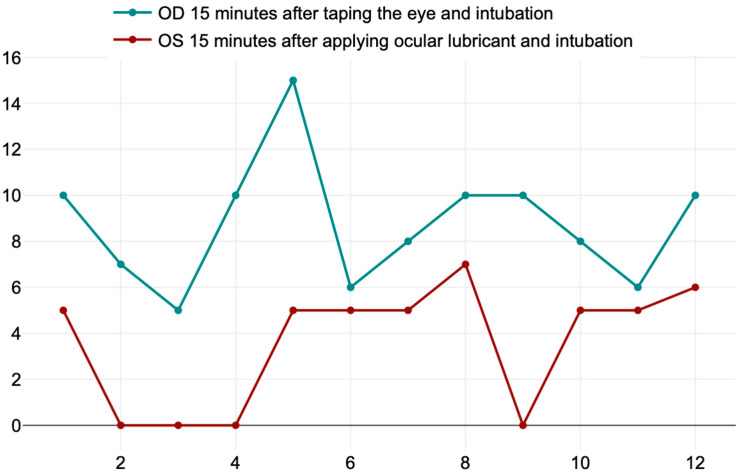
Values of the Schirmer Tear Test in the right (OD) and left eye (OS).

There was a *significant* decrease in tear secretion value in sheep who received only the ocular lubricant compared with the taped eye (Figure 7). Our hypothesis from our study is that closing the eyelid in the right eye (OD) during anesthesia prevents a rapid evaporation of the tear film, and a better outcome which was also seen in the values that we obtained.

**Figure 8:**
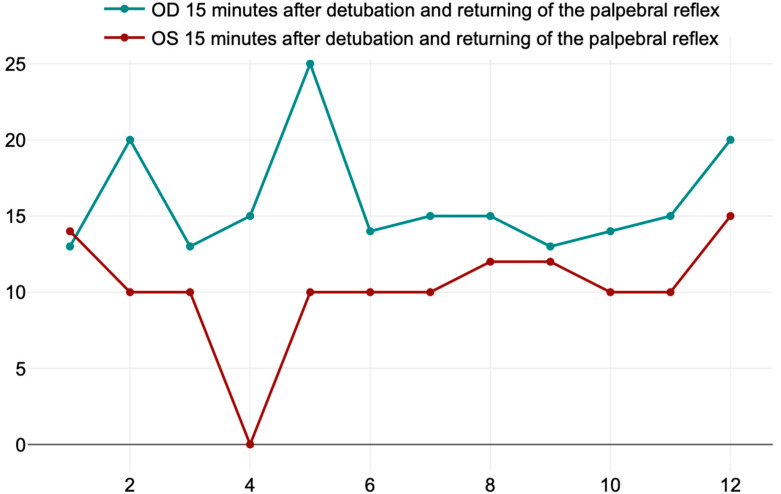
Values of the Schirmer Tear Test in the right (OD) and left eye (OS) 15 min after detubation and returning of the palpebral reflex.

The mean values ± SD of the right (OD) and left eye (OS) after 15 min of extubation and returning of the palpebral reflexes were, respectively, 16 ± 3.71 mm/min and 10.25 ± 3.67 mm/min. There was a *significant* difference regarding the return of tear production (T_3_) in the right eye (OD), which was taped, compared with the left eye (OS), which received only two drops of ocular gel with 1% hyaluronic acid. The *t*-value is 3.81253, and the *p*-value is 0.000476. The result is *significant* at *p* < 0.05 (Figure 8).

**Figure 9:**
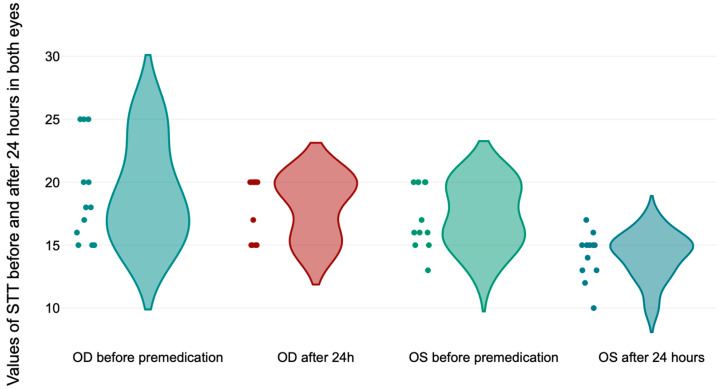
Values of the Schirmer Tear Test in both eyes before premedication and 24 h after premedication.

After 24 h (T_4_), we evaluated the return of the tear production values at baseline. In the right eye (OD), which was taped during anesthesia, the results of the paired-*t* test indicate that there is a *non-significant* small difference between the values of the STT before premedication (*M* = 19.08, *SD* = 3.96) and 24 h after premedication (*M* = 18.03, *SD* = 2.42), *t*(11) = 1.1, *p* = 0.137, compared with the values for the left eye (OS), who received the ocular lubricant where the results of the paired-*t* test indicated that there is a significantly large difference between the values of the STT before (*M* = 17.33, *SD* = 2.53) and after 24 h from premedication (*M* = 14.16, *SD* = 1.89), *t*(11) = 4.4, *p* < 0.001 (Figure 9).

At the end of the study, each sheep had a Fluoresceine test (AIESI^®^ Fluorescein Sodium Fluorescein Sterile Ophthalmic Strips for Eye Tone Test HOSPIFLUO STRIPS, Manufactured by CONTACARE OPHTHALMICS & DIAGNOSTICS, Gujarat, India) performed, achieving a negative result which established that none of them had any corneal abrasions and none were displaying other clinical symptoms like blepharospasm, ocular pain, and epiphora that could indicate an affected cornea.

All the mean values ± SD we obtained are displayed in the following table (Table 1).

## 4. Discussion

Based on the findings of this study, the results suggest a decrease in tear production during anesthesia with midazolam, butorphanol, ketamine, and the other results suggest that closing the eyelid in the right eye (OD) during anesthesia prevents a rapid evaporation of the tear film and a fast return to normal values of STT compared with the left eye (OS), where we only used two drops of the ocular lubricant.

To our knowledge, the results of this study are not comparable within the literature, as there are no other studies that have evaluated the impact on tear production after the administration of this sedative agents nor the impact of taping the eye compared with instilating the ocular lubricant with 1% hyaluronic acid in the other eye, in Tsurcana sheep.

The mean baseline values of the STT (T_0_) before premedication were, respectively, 19.08 ± 3.96 mm/min in the right eye (OD) and 17.33 ± 2.53 mm/min in the left eye (OS). Previous research has indicated that the average tear secretion (TS) rates in various sheep breeds are as follows: Sanjabi sheep have a baseline rate of 18.52 ± 2.55 mm/min, as reported by Ghaffari et al. [30]; Romanov sheep have a lower baseline rate, with 11.59 ± 4.07 mm/min [31]; and Merinos sheep have a higher rate of 18.80 ± 1.82 mm/min [32]. Variations in filter papers, the positioning of strips in the conjunctival sac, and the person conducting the test can influence TS values due to differences in absorptive capacities, as noted by Ghaffari et al. [30] and Rothschild et al. [33]. Moreover, a previous study carried out on horses stated that the amount of TS can vary according to the season [27]. All our data were obtained in the spring season, at the same period of day, from 9 am to 3 pm; therefore, changes in the daily cycle and season, which might influence the test results, most likely did not affect our STT results since the differences between STT values were observed in readings from different seasons, and early in the morning or in the late evening [4,24,28,29].

### 4.1. Effects of the Anesthetic Drugs on Tear Production

The precise mechanism behind the reduction in TS in sheep remains uncertain. However, a prior investigation revealed that the intramuscular administration of a combination of sedatives and opioid led to a decrease in TS levels in dogs [17]. In our study, the use of opioids, sedatives, and dissociative anesthetics led to a fast and constant decrease in the tear secretion following the premedication, induction, and maintenance phase.

Dodam et al. [17] proposed that the reduction in TS levels could be attributed primarily to one or a combination of the following mechanisms: the central effects of these drugs on the autonomic regulation of tear production, effective pain relief, vasoconstriction of the tear gland, and alterations in metabolism at the cellular level of the gland. Additionally, another potential explanation for the decrease in TS levels could be that the administration of sedatives resulted in a reduced blinking rate and increased evaporation of tears, as suggested by Leonardi et al. [34].

### 4.2. Effect of 1% Hyaluronic Acid Compared with the Eye Closed

Although sodium hyaluronate positively impacts tear film stability, a recent pilot study revealed that among two groups treated with hyaluronic acid (HA), the group receiving a lower concentration (0.25%) had higher STT readings compared to the group receiving a higher concentration (1%) [35]. In our study, closing the eyelid in the right eye (OD) during anesthesia prevented a rapid evaporation of the tear film and a fast return to normal values of STT compared with the left eye (OS), where we only use the ocular lubricant with 1% hyaluronic acid. While ocular lubricants do not increase tear production rates, their use is recommended to reduce corneal dehydration, enhance corneal wettability, and protect the cornea [36].

## 5. Conclusions

The data obtained in the present research provide important information, helping the anesthesiologists to better manage the effect of anesthetic drugs regarding tear production. Anesthesia is required for several surgery procedures, and a good understanding of how the drugs affect the STT and how the cornea can be protected is important for good perianesthetic management. This study suggested that the protocol used with midazolam, butorphanol, and ketamine decreases tear production shortly after intramuscular administration in sheep. There are numerous methods available to protect the cornea, but the one that we studied by taping the eyelid of the right eye (OD) and putting ocular lubricant with 1% hyaluronic acid in the left eye (OS) suggested better STT values and a faster return to baseline after the palpebral reflexes appeared in the right eye (OD) compared with the left one (OS).

## Figures and Tables

**Table 1:** Values of mean and standard deviation of tear production in both eyes during different periods in time.

	STT Values in Both Eyes at Different Time Periods	Mean ± SD
T_0_	OD before premedication	19.09 ± 3.96 mm/min
	OS before premedication	17.33 ± 2.53 mm/min
T_1_	OD 15 min after premedication	13.08 ± 3.31 mm/min
	OS 15 min after premedication	12 ± 3.43 mm/min
T_2_	OD 15 min after taping the eye and intubation	8.75 ± 2.70 mm/min
	OS 15 min after instilating the ocular lubricant and intubation	3.58 ± 2.71 mm/min
T_3_	OD 15 min after detubation and returning of the palpebral reflex	16 ± 3.71 mm/min
	OS 15 min after detubation and returning of the palpebral reflex	10.25 ± 3.67 mm/min
T_4_	OD after 24 h	18.08 ± 2.42 mm/min
	OS after 24 h	14.16 ± 1.89 mm/min

## Data Availability

The data generated in this study are presented in the tables of this article. For any further information, the reader can contact the authors.
